# Retinal Tacks for Complicated Retinal Detachment: Retinal Tacks in the Times of Modern Small-Gauge Vitrectomy

**DOI:** 10.1155/2022/1968434

**Published:** 2022-03-31

**Authors:** Luca Mautone, Simon Dulz, Christos Skevas, Maximilian Schultheiss, Martin Stephan Spitzer

**Affiliations:** Department of Ophthalmology, University Medical Center Hamburg-Eppendorf, Hamburg, Germany

## Abstract

**Purpose:**

To investigate the efficacy and safety profile of retinal tacks (RTs) in cases of retinal detachment (RD) with advanced proliferative vitreoretinopathy (PVR).

**Materials and Methods:**

In this single-center, retrospective study medical record, optical coherence tomography and ultra-widefield fundus images of patients with complex PVR-related and RT surgery were reviewed. All cases underwent 23G pars plana vitrectomy (PPV), RT implantation, retinectomy, circumferential intraoperative laser retinopexy, and silicone oil tamponade.

**Results:**

Fourteen eyes of 14 patients with complex rhegmatogenous RD with PVR were included: 7 cases showed PVR grade C type P and 7 combined grades A and P. RTs were positioned at contracted, stiffened retinal areas to achieve attachment of retinectomy borders after extensive PVR peeling. Patients underwent on an average of 1.3 PPVs (range 0–3) prior RT surgery. An average of 2.5 RTs (range 1–4) were implanted. Only in a single eye, a recurrent RD occurred. In 10 eyes, the silicone oil tamponade was still in place at the last follow-up. In 5 eyes, the silicone oil could be removed without redetachment in all of these cases (average of 31.3 weeks, range 11.4–53). No RT-related intraoperative or postoperative complications like dislocation or bleedings were observed.

**Conclusion:**

RTs have the potential to improve the treatment of complex PVR-associated RD. RT can be a useful surgical tool to reattach borders of retinectomies in advanced PVR. No RT-associated complication were observed in this study.

## 1. Introduction

In 1983, Ando and Kondo proposed the permanent implantation of plastic retinal tacks (RT) to fixate everted flaps of giant retinal tears [1]. Later, two other RT models—one made of stainless steel and the other of titanium alloy—were developed to treat complicated retinal detachments [2, 3]. Such tacks showed to be useful to counteract retinal traction in complex cases, for example, for fixating a stiff and shrinking retina of retinectomy borders or large retinal tears. RT was also employed to unfold giant retinal tears in the presence of not removable traction. Moreover, the use of RT was not limited to the primary treatment of rhegmatogenous retinal detachment (RD) but also expanded to other severe conditions, for example, in cases of recurrent RD due to proliferative retinopathy (PVR), perforating trauma, giant retinal tear, and tractional RD in advanced proliferative diabetic retinopathy [3–11]. In the lack of evidence for ocular toxicity [5, 9, 10, 12, 13], both the permanent as well as the temporary implantations of RT to unfold and reattach the retina during surgery were described [2, 3, 5, 8, 11]. RTs were rarely associated with severe postoperative complications [2, 3, 5]. Despite this, subretinal or vitreous bleeding after tack implantation and several other complications associated with dislocated tacks were reported [8].

Although mechanical fixation of RD with RTs has greatly diminished due to the adoption of new surgical tools, such as heavy liquids or endolaser retinopexy, RTs found new utility in anchoring epiretinal electrode arrays [14, 15]. As epiretinal prosthesis is not manufactured any longer, RTs today are hardly used by vitreoretinal surgeons. Sometimes a not reattachable shrinking or stiff retina is still encountered despite meticulous peeling, additional episcleral surgery, or even heavy liquid injection. In these situations, RTs may remain an effective surgical tool. The literature related to the clinical use of RT is limited and has been mostly published decades ago before the introduction of important innovations in vitreoretinal surgery. Thus, the aim of this study was the investigation of the efficacy and safety profile of RT in complicated PVR-related RD in the context of modern vitreoretinal surgery.

## 2. Material and Methods

This retrospective study followed the tenets of the Declaration of Helsinki. Medical records from the electronical database at the Department of Ophthalmology of the University Medical Center Hamburg-Eppendorf were reviewed. The data were anonymized before analysis. Complicated cases of rhegmatogenous RD with RT implantation were included. Other etiologies like tractive RD due to proliferative diabetic retinopathy, trauma-related RD, or further RD etiologies were excluded from this study. RTs were not removed for the whole follow-up period.

Patient records and imaging with ultra-widefield confocal scanning ophthalmoscopy (Optos California, Optos GmbH, Düsseldorf, Germany), swept-source optical coherence tomography (SS-OCT) (Topcon Triton, Topcon GmbH, Willich, Germany), and fundus stereo photography (Topcon Triton, Topcon GmbH, Willich, Germany) were retrieved from the electronic medical records.

Two experienced vitreoretinal surgeons—M.S.S. and M.S.—performed the surgeries. All patients had at least 4 inferior clock hours of PVR-C prior to RT implantation.

In the informed consent for surgery, patients were informed about the off-label use of this product and agreed with its use. Patients underwent a 23G pars plana vitrectomy with retinectomy, circumferential laser retinopexy, peeling of the PVR membrane, 5000cs silicone oil tamponade, and RT implantation. RTs were implanted at the edges of retinectomies that were still contracted despite meticulous peeling of PVR membranes and 180° peripheral retinectomy. Laser treatment was applied in a circular pattern around the margins of the RTs.

**Figure 1:**
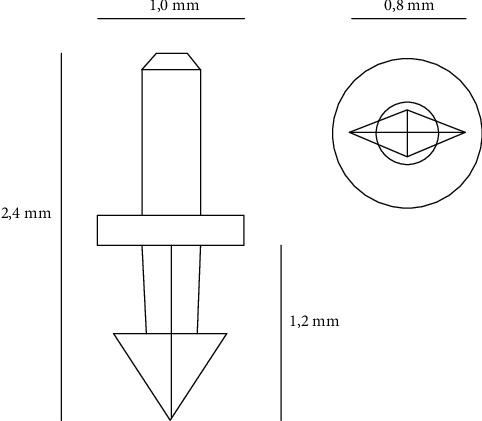
Schematic representation of the retinal tack. On the right side: lateral view of the tack. On the left side: inferior view of the tack.

Titanium retinal tacks (Heinmann model, G-33437(*t*), Geuder AG, Heidelberg, Germany) were placed. A schematic representation of the tacks is shown in Figure 1. The length of this prothesis is 2.4 mm and the maximum diameter 1.0 mm. The titanium RT have an arrow shape with a single sharp peak, and the shaft is divided by a round slice in the middle. RTs mounted on an applicator forceps are placed through a 20G sclerotomy (enlargement of the initial 23G sclerotomy was performed in all cases) and then pushed through the retina into the sclera ensuring that only the sharp peak perforated the retina. The RT was placed into areas in which the retina remained stiffened or contracted, despite extensive peeling of PVR membranes. Moreover, contracted retinal flaps of giant tears or retinectomy edges could be flattened and reattached by RT implantation. This procedure was performed under perfluorocarbon liquid ([PFCL], F-Decalin, Fluoron GmbH, Ulm, Germany) (Geuder AG, Heidelberg, Germany). At the end of every case, 5000cs silicone oil was injected into the vitreous space. For statistical analysis, a *t*-test was used with significance levels set at *p* > 0.05.

## 3. Results

Twenty-one patients who underwent RT surgery were identified between January 2017 and March 2021. Two patients were excluded because they did not attend any follow-up visits after surgery.

For this study, 14 eyes of 14 patients with complicated PVR-associated retinal detachment with a mean follow-up of 74 weeks were analyzed (range 4–180 weeks). Patients' characteristics are summarized in Table 1. Encircling band surgery or a phacoemulsification with intraocular lens implantation was performed prior to PPV in 5 and 12 patients (36% and 86%), respectively.

Prior to RT surgery 12 of 14 eyes (86%) underwent a surgical intervention and on average had 1.3 PPVs before they underwent PPV with RT implantation (range 0–3, mode 2 PPV). A macular involvement of the RD was seen in 11 eyes (79%), and the average number of detached quadrants of the retina was 3.4 (range 2‐4, mode 2 quadrants). As shown in Table 2, all patients had a PVR grade C, with PVR of anterior type or combined type (anterior and posterior) in 50% of cases, respectively.

The indications for RT are summarized in Table 3. These were fixing the edge of relaxing retinectomy due to retina stiffness or traction (*n* = 8), fixing the edge of a relaxing retinectomy with traction due to not removable PVR membranes (*n* = 5), and unfolding a giant retinal tear border with traction (*n* = 1). Ten eyes were pseudophakic and 1 aphakic. Phacoemulsification with intraocular lens implantation was performed in 1 eye during RT surgery.

A mean of 2.5 RTs (range 1–4, mode 2 RT) were implanted. In 4 (29%) of the cases, RT were needed to archive retinal reattachment despite encircling band implantation prior to PPV in the same surgery. Tacks were placed under PFCL in all eyes except patient 4 (see Table 2). Retinal reattachment could be achieved in every eye after RT surgery, and no eversion of retinectomy or tear borders was seen in this series.

Due to mental disability, the best correlated visual acuity (BCVA) was not measurable in 1 patient. The average BCVA improved from a mean preoperative visual acuity of 1.6 (range 2.7–0.4 logMAR) to 1.56 logMAR (range 2.7–0.4 logMAR) at the end of the follow-up (*p*=0.42). Six patients experienced an improvement and 5 a worsening of BCVA (46% and 39%, respectively) by the last control. In 2 eyes, visual acuity remained unchanged (15%). Eleven patients (79%) underwent additional surgical interventions after the RT surgery. Silicone oil removal, which was not counted as additional surgical procedure, was performed in 5 patients (36%) after a mean of 54 weeks (range 31‐75 weeks). In 4 eyes, the silicone oil removal was uncomplicated. In 1 patient (patient 3), silicone oil injection was performed due to postoperative prolonged hypotony. However, no recurrent RD was observed after silicone oil removal. The average follow-up after the removal of silicone oil was 31.3 weeks (range 11.4–53 weeks), whereas endotamponade could be removed in selected cases in which RTs were not extracted.

**Figure 2:**
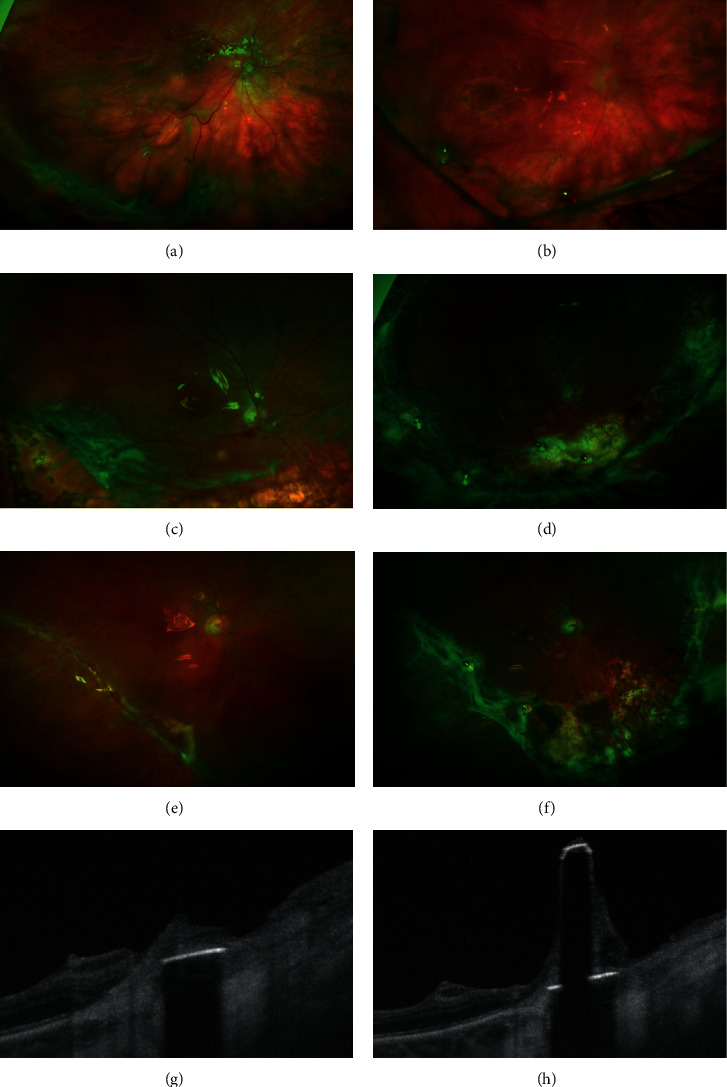
Ultra-widefield scanning laser ophthalmoscopy. Fundus photography of patient 1 before surgery (a) and 5 weeks after surgery (b). 2 retinal tacks (RT) are placed at 6 o'clock and 7 : 30 positions. A fibrous reaction at the RT situs and along the retinectomy is seen. The retinectomy borders are not detached, and the fibrous strands are held by RTs. Fundus photography of patient 3 before surgery (c) and 6 months after surgery (d). 4 RTs are placed at 5 : 30, 6, 7, and 8 o'clock positions. A fibrous reaction involves RTs and retinectomy borders. Fundus photography of patient 8 before surgery (e) and 11 months after surgery (f). 2 retinal RTs were placed at 6 : 30 and 8 : 30 positions to reattach the stiff retina of the retinectomy border close to the macula. Retinectomy borders are not everted after surgery, despite fibrous reaction (b, d, f). Swept-source optical coherence tomography (SS-OCT) scans with segmentation of the RT in patient 11 (g, h). SS-OCT of the RT shows a fibrous reaction around the tack and on the tack's shaft.

Postoperative findings and complications are summarized in Table 4. In 80% of the eyes (8 of 10), a nontractional fibrous reaction around the RTs was observed (see Figure 2).

For example, an OCT scan of a retinal tack performed in patient 16 shows the presence of fibrotic material around the tack and on its shaft (see Figure 2). The surrounding retinal tissue is not detached but appears atrophic and fibrotic. In 71% (5 of 7 eyes), a fibrous reaction along the retinectomy borders was noted (see Figure 2). In these cases, a straight strand of proliferating tissue along the retinectomy borders connecting RTs was observed. The fibrous reaction evolved over time without extending centrally to the retinectomy borders. Despite this peripheral fibrotic tissue, the retinectomy borders and the retina were not detached. It seems that the tractional forces of the fibrotic strands were well controlled by the RT (see Figure 2).

A recurrent RD with macula involvement due to PVR occurred in one patient 53 days after surgery (patient 2). In this patient, with a history of severe posterior uveitis of unclear origin, the recurring RD came from the opposite side of the primary RD where no RTs were in place.

No RT dislocation or any other RT-related complications such as hemorrhage occurred in this series.

Several postoperative complications were observed after RT surgery so that a further surgical intervention or vitreoretinal surgery were performed (43% and 21%, respectively, see Table 3).

## 4. Discussion

We report the long-term safety and efficacy of RT in the treatment of complicated RD with PVR. RT enables retinal attachment in cases of irremovable tractions and stiff retina allowing anatomical success. Postoperatively, tacks withstand postoperative PVR reactions preventing recurrent RD.

Despite important improvements in the surgical techniques and vitrectomy technologies, several challenging situations are still difficult to solve, despite important progresses in vitreoretinal surgery. Some of these are the presence of persistent tractions due to nonremovable retinal membranes or a stiff and shrinking retina due to structural shortening and contraction. The treatment of these anatomical issues is necessary since they contrast the mechanisms enabling retinal reattachment according to the Custodis' principle, namely, retinal approximation and fixation [16]. Approximation of the detachment is enabled by the endotamponade, while laser photocoagulation induces a bond between the retina and the retinal pigment epithelium. Yoon and Marmor [17] and other authors [18,19] demonstrated that the retinochoroideal adhesion of the detached retina already increases 24 hours after laser photocoagulation. As normal adherence is restored about 3 days after laser retinopexy [20], a surgical technique with the immediate onset of efficacy is required to counteract persisting retinal traction or reattach the contracted retina. Our results suggest that RTs are a safe and effective solution for otherwise not removable retinal tractions. This implant provides an immediate attachment and fixation of the retina in complicated cases of not only primary RD but also recurring RD.

PVR is the commonest cause of recurring RD with 93%, after relaxing retinectomy [21]. All eyes of this study presented with PVR grade ≥ C and most of them underwent multiple RD surgeries, both risk factors for final anatomical failure [22]. Considering these data, the use of RT suggests an improvement of the surgery's outcomes in such very complicated cases. Only one redetachment after RT surgery occurred and originated from the opposite side of the RD treated with retinectomy and RTs. Moreover, this patient had a severe uveitis that was treated with azathioprine as an ocular comorbidity. In spite of this promising morphological results, BCVA did not improve significantly. This is explained not only by the previous surgeries but also by the complicated situation encountered. Therefore, given the fact that BCVA deteriorates with every additional redetachment [23], RT can prevent further visual deterioration.

Fibrosis along retinectomy edges is the most frequent cause of recurring RD after retinectomy [21]. Postoperatively, the development of a fibrotic reaction spanning between retinal tacks and along the retinectomy borders was also noted in our series as previously observed in several series with complex RD different etiology [4–6, 8]. This reaction increased over time and exercised traction on tacks, retina, and retinectomy borders. However, the RT is able to anchor the retina into the sclera and directly resist against persistent tractional forces. RTs could counteract tractions and prevent a further PVR-associated retinal detachment.

Fibrotic tissue did not only grow along retinectomy borders but also surrounded RTs as reported after inserting titanium alloy and stainless steel RTs [3–5, 8]. Raster electron microscopy revealed macrophages on the surface of a dislodged tack without detecting a further membranous structure [8]. In a rabbit model, titanium tacks were surrounded by a proliferation of avascular scar tissue. The tissue appeared within a month after implantation, and histological examination showed it to be a proliferation of retinal glial cells in proximity of the retina and fibroblastic cells arising from the choroidea at the level of the sclera. Around RTs, the retinal architecture and photoreceptors were not preserved [7]. Histopathological findings in one enucleated eye confirmed the findings of the animal model and showed retinal atrophy of the RT surrounding area [12]. We found several correlations to these previously described histopathological findings in OCTs of RT (see Figure 2). First of all, OCT demonstrated the presence of hyperreflective material around the shaft of RTs. Biomicroscopically, the fibrous reaction correlated with the material on the shaft. Alike histological studies, the retinal architecture, photoreceptor, and also retinal pigment epithelial layer appeared atrophic surrounding the implant. Due to obscuration of the retinal structure caused by the metal alloy, the in vivo determination of the status of the retina directly under the retinal tack is not possible. OCT demonstrated a homogenous, hyperreflective membrane originating from the choroid and spreading around the tack. This appearance suggests the presence of a scarring reaction originating from the choroid between the retina and the RT.

The incidence of redetachment following silicone oil removal is 6.9–36% in eyes with retinal detachment and PVR [24–27]. In this series, silicone oil was removed in 5 eyes and no recurring RD occurred during the whole follow-up.

The removal of retinal tacks is possible and without significant complications. De Juan et al. [5] removed 74 stainless steel RTs in 23 cases of retinal detachment and reported bleeding into the subretinal space as the most common complication, which could be easily controlled intraoperatively with diathermy, aspiration, and by increasing intraocular pressure. More recently, the complication-free removal of the RT used for fixation of epiretinal electrode arrays as part of implanted visual prostheses was reported [15]. Differently, in this series, RTs were not removed in order to preserve the counterpointing effect of tacks against retinal traction.

No signs of ocular toxicity due to the intraocular presence of titan alloy were observed for the whole follow-up. Previous investigations performed in a RD rabbit model showed no histological or electrophysiological evidence of diffuse injury due to toxicity one year after treatment with titan alloy RTs [7]. Similarly, no signs of ocular toxicity were described in a cohort of patients with titanium alloy RTs due to fixation of electrode arrays for visual prosthesis after prolonged implantation up to 19 months [15]. In cases of titanium alloy RTs adopted for RD treatment, a case report with a follow-up of 10 years after bulbus perforation suggests a good long-term intraocular tolerability of titanium alloy tacks [9]. Furthermore, the lack of toxicity of stainless steel RTs for giant retinal tear during a follow-up of 21 years was reported [10].

Complications related to tacks are well described in the literature. Intraoperative, subretinal hemorrhages at the side of penetration and slippage of tacks are reported [4]. Tangential forces of the fibrotic reaction around RT and slanting placement with incomplete penetration of the retina, choroid, and also sclera are potential causes of instability of this retinal implant [8]. As a consequence, displacement or dislodgement of RD brings to vitreous hemorrhage, retinal phlebitis, and chorioretinal atrophy, till migration into the anterior chamber with consequent corneal edema or even retinal detachment [5, 8, 28]. In this series, no adverse intraoperative or postoperative events connected to RTs were observed. There are several reasons for the lack of complications in this series. First of all, other than in other publications, the use of heavy liquids during the RT placement enables the surgeons to easily place RT perpendicularly into the sclera through an already reattached and outspread retina achieving an upright penetration of tacks into the retina and the sclera. Another reason is the design of the tacks: the stabilization of the Heimann tack model is facilitated by the spike design, a missing element in different RT models used by other groups [2, 3]. Indeed, in a series of extruded RTs, all the dislodged tacks were without a spike [8].

The retrospective nature and the limited case number of this study are surely remarkable limitations. Furthermore, the heterogeneity of eyes needing this further surgery is challenging.

With continuous surgical innovation in vitreoretinal surgery, the use of RTs declined over the past decades. As a consequence, manufacturers discontinued the production of this device. Recently, investigations describing the optimization of the RT design in order to resist major tractions were published [29], leaving place to speculations concerning a return of RT into broad clinical use. Even if this product is not labeled for the clinical use, we believe that RT remains a valuable surgical tool for the most complicated situation, for example, massive retinal shortening, and they can easily be combined with modern vitreoretinal surgical techniques. In summary, we outline the value of RTs in selected cases of complex RD with PVR. RTs might be an effective tool to achieve retinal attachment in cases of not attachable shrinking or stiff retinas, especially when retinectomy or an encircling band is not successful. Furthermore, RTs counteract traction if membranes are not removable and prevent recurrent RD in cases of severe PVR reaction along retinectomy borders. In our series, no RT-related complications or toxicity signs were observed after a long follow-up.

## Figures and Tables

**Table 1:** Patients' surgery data.

*Gender (n)*	
(i) Male	6 (42%)
(ii) Female	8 (58%)
Age (years)	64.6 (range 10–84)

*Eye*	
(i) R	10 (71%)
(ii) L	4 (29%)
Follow-up (weeks)	74 (range 4–180)

*Visual acuity (logMAR)* ^*†*^	
(i) Preoperative	1.60 (range 2.7–0.4)
(ii) At the end of follow-up	1.56 (range 2.7–0.4)
(iii) Improvement	6 (46%)
(iv) Worsening	5 (39%)
(v) Unchanged	2 (14%)

*Lens status (n)*	
(i) Phakia	2 (14%)
(ii) Pseudophakia	11 (79%)
(iii) Aphakia	1 (7%)
Prior vitrectomies (*n*)	1.3 (range 0–3, mode 2)

*PVR grade (n)*	
(i) C a + p	7 (50%)
(ii) C p	7 (50%)

*Macular involvement (n)*	
(i) On	3 (21%)
(ii) Off	11 (79%)

Involved retinal quadrants (*n*)	3,4 (range 2–4, mode 2)
*Indication for RT (n)*	
(i) Fixing the edge of relaxing retinectomy due to retina stiffness or traction	8 (57%)
(ii) Fixing the edge of relaxing retinectomy with traction due to not removable PVR	5 (36%)
(iii) Unfolding giant tear border with traction	1 (7%)

Placed RT (*n*)	2.5 (range 1–4, mode 2)
Encircling band before implantation of RT (*n*)	5 (36%)
Retinectomy (*n*)	
(i) 180°	9 (64%)
(ii) >180°	2 (14%)
(iii) 360°	2 (14%)
(iv) Not specified	1 (7%)

Use of PFCL (*n*)	13 (93%)
Silicone oil removal (*n*)	5 (36%)
(i) Time after surgery (weeks)	54 (range 31–75)
Fibrous reaction at RT (*n*)	8 (80%)
Fibrous reaction along retinectomy borders (*n*)	5 (71%)
Patients with complications after surgery with RT (*n*)	12 (86%)
Recurring retinal detachment (*n*)	1 (7%)
Patients undergoing further surgical intervention after surgery with RT (*n*)	11 (79%)
*Following interventions (mean, % of patients)*	
(i) Overall^‡^	1.28 (range 0–6, mode 1), 43%
(ii) Vitreoretinal surgery^‡^	0.36 (range 0–3, mode 1), 21%

^†^No significant improvement of the visual acuity (*p*=0.42). ^‡^Silicon oil removal is not included. RT = retinal tacks.

**Table 2:** General data of patients.

Patient	Sex/age	Side	Ocular comorbidities	Previous PPV (*n*)	PVR grade and type	Quadrants involved by detachment	Macular involvement	Preoperative BCVA (logMAR)	BCVA at the end of follow-up (logMAR)
1	W/81	R	Aphakia	2	C, p	2	Off	0.8	2.2
2	M/56	R	Posterior uveitis, complicated phacoemulsification with IOL implantation, and posterior capsule rupture	0	C, a + p	2	Off	2.2	2.2
3	M/84	R		1	C, p	2	Off	2.7	2.2
4	M/61	R		2	C, p	2	On	1.3	0.8
5	W/82	R	Perforating keratoplasty by herpetic keratouveitis	0	C, a + p	4	On	2.7	2.7
6	M/63	R		0	C, a + p	4	Off	2.2	0.4
7	M/77	L		3	C, a + p	2	Off	1.3	0.7
8	W/10	R	Retinopathia praematorum	0	C, p	4	Off	1.4	2.2
9	W/75	R		1	C, p	4	Off	1.8	1
10	M/62	L		2	C, p	2	On	0.4	1.3
11	W/57	R		2	C, p	2	Off	0.5	0.6
12	M/70	L		2	C, a + p	2	Off	1.4	1.3
13	W/77	R		2	C, a + p	4	Off	2.2	2.7
14	M/50	L		0	C, a + p	4	Off	NP	NP

M = male; F = female; A = PVR anterior type, P = PVR posterior type; NP = not possible.

**Table 3:** Indications and surgical procedures.

Patient	Intraoperative findings	Retinal tacks (*n*)	PFCL	Retinectomy (°)	Celcalge	Lens status before surgery with retinal tacks	Phaco with IOL implantation in surgery with retinal tacks	Operateur
1	Fixing the edge of relaxing retinectomy with traction due to not removable PVR	2	+	180°	+	AP	−	1
2	Unfolding giant tear border with traction	3	+	180°	−	PP	−	2
3	Fixing the edge of relaxing retinectomy due to retina traction	4	+	240°	−	PP	−	2
4	Fixing the edge of relaxing retinectomy due to retina stiffness	3	−	180°	−	PP	−	2
5	Fixing the edge of relaxing retinectomy with traction due to not removable PVR	2	+	270°	−	PP	−	2
6	Fixing the edge of relaxing retinectomy due to retina stiffness	2	+	180°	−	P	+	2
7	Fixing the edge of relaxing retinectomy due to not removable PVR	3	+	Focal (° not described)	−	PP	−	2
8	Fixing the edge of relaxing retinectomy due to retina stiffness	4	+	° not described	+	P	-	2
9	Fixing the edge of relaxing retinectomy with traction due to not removable PVR	2	+	180°	−	PP	−	2
10	Fixing the edge of relaxing retinectomy due to retina stiffness	3	+	180°	−	PP	−	2
11	Fixing the edge of relaxing retinectomy due to retina traction	1	+	Focal (° not described)	+	PP	−	2
12	Fixing the edge of relaxing retinectomy due to retina stiffness	1	+	Focal (° not described)	+	PP	−	2
13	Fixing the edge of relaxing retinectomy with traction due to not removable PVR	2	+	360°	+	PP	−	1
14	Fixing the edge of relaxing retinectomy due to retina stiffness	3	+	360°	−	P	Lentectomy	2

AP = aphakia; PP = pseudophakia; P = phakia; PVR = proliferative vitreoretinopathy; PFCL = perfluorocarbon liquid; Operateur 1 = M.S; Operateur 2 = M.S.S.

**Table 4:** Complications and additional interventions.

Patient	Follow-up (days)	Retinal detachment after surgery with retinal tacks	Silicon oil removal (days after surgery)	Fibrous reaction at RT	Fibrous reaction between RTs	Eversion of retinectomy/tear borders	Complications	Further surgeries
1	54	−	−	+	+	−	Persisting corneal erosion and macular atrophy	(i) Amniotic membrane transplantation due to persisting corneal erosion
2	674	+	−	−	−	−	IOL subluxation, posterior synechiae, macular atrophy, macular edema, and PVR-related recurring retinal detachment	(i) PPV, IOL removal, PVR peeling, laser cerclage, and 5000cs silicone oil (ii) Subtenon triamcinolone injection due to macular edema
3	597	−	226	+	+	−	Macular edema, epiretinal gliosis, chronic hypotony, retinal folds, and silicone oil migration into the anterior chamber	(i) Phacoemulsification with IOL implantation (ii) Silicone oil removal with membrane peeling (iii) 5000cs silicone oil injection due to hypotony with retinal folds (iv) 2x Healon injection in the anterior chamber (v) 5000cs silicone oil injection and anterior chamber washout due to hypotony and silicone oil migration into the anterior chamber
4	309	−	−	+		−	Macular edema, epiretinal gliosis, IOP elevation, and PVR	
5	327	−	−			−	Optic nerve atrophy, graft failure, and secondary glaucoma	(i) Suture removal after penetrating keratoplasty
6	585	−	505	+		−	IOP elevation, anisometropia, recurring corneal erosion, and PVR	(i) Silicone oil removal, anterior chamber washout, and posterior capsule dissection
7	822	−	528	+	−	−	Chronic macular edema	(i) Silicone oil removal (ii) 1x subtenon triamcinolone injection and 4x dexamethasone intravitreal implant
8	562	−	−			−	Cataract and PVR	(i) Lentectomy with IOL implantation
9	349	−	−	+	+	−	PVR and macular edema	(i) PPV, silicone oil change, PVR, and ILM peeling
10	357	−	216	+	+	−	PVR	(i) Silicone oil removal
11	618	−	409	−	+	−		(i) Silicone oil removal
12	1260	−	−	+	+	−	Anisometropia and macular atrophy	(i) Add-on IOL implantation
13	696	−	−			−	PVR and rolled-up central retina	
14	53	−	−					

ILM = internal limiting membrane; IOL = intraocular lens; PPV = pars plana vitrectomy; PVR = proliferative vitreoretinopathy; RT = retinal tack.

## Data Availability

All the results and rough data of this study are included in the manuscript.
